# Risk factors for postoperative recurrence in patients with stage II colorectal cancer

**DOI:** 10.1186/s12885-023-11093-w

**Published:** 2023-07-14

**Authors:** Zhi-Zhong Xiong, Ming-Hao Xie, Xian-Zhe Li, Long-Yang Jin, Feng-Xiang Zhang, Shi Yin, Hua-Xian Chen, Lei Lian

**Affiliations:** 1 Department of Gastrointestinal Surgery, Department of General Surgery, the Sixth Affiliated Hospital, Sun Yat-Sen University, 26 Yuancun Er Heng Rd., Guangzhou, 510655 Guangdong China; 2 Department of Gastrointestinal Surgery, Department of General Surgery, Guangdong Provincial Key Laboratory of Colorectal and Pelvic Floor Diseases, the Sixth Affiliated Hospital, Sun Yat-Sen University, Guangzhou, China; 3 Department of General Surgery, the First Affiliated Hospital of Nanchang University, Nanchang, China

**Keywords:** Colorectal cancer, Predictive factors, Recurrence, Stage II

## Abstract

**Background:**

Recurrences are the main reasons for unfavorable outcomes for patients with stage II colorectal cancer (CRC). To obtain a clear understanding of the high-risk factors, further investigation is warranted. The present study aimed to analyze the risk factors associated with postoperative recurrence in patients with stage II CRC.

**Methods:**

Eligible patients with pathologically confirmed stage II CRC were enrolled in the study retrospectively based on a prospectively maintained database from April 2008 to March 2019. The Kaplan–Meier method were used to calculate the overall survival (OS) rate and the cumulative recurrence rate. Univariate and multivariable Cox regression analyses were performed to identify risk factors for recurrence.

**Results:**

There were 2515 patients included, of whom 233 (9.3%) developed local or distant recurrence. Recurrence was associated with a significantly worse 5-year OS (45.4% *vs*. 95.5%, *p* < 0.0001). The 5-year cumulative recurrence rate was 13.0% in patients with stage II CRC. On multivariable Cox analysis, tumor size (Hazard Ratio (HR) [95% confidence interval (CI)] = 1.79[1.38, 2.33]), preoperative carbohydrate antigen (CA) 125 level (HR [95% CI] = 1.78[1.17, 2.70]), preoperative CA 199 level (HR [95% CI] = 1.56[1.09, 2.22]), and ulcerating tumor (HR [95% CI] = 1.61[1.19, 2.17]) were found to be associated with postoperative recurrence. Adjuvant chemotherapy was associated with a lower cumulative recurrence rate in patients with these risk factors (*p* = 0.00096).

**Conclusion:**

The tumor diameter, preoperative CA125 level, preoperative CA199 level, and an ulcerative tumor can predict postoperative recurrence in patients with stage II CRC, and postoperative chemotherapy could reduce the cumulative recurrence rate in patients with these high-risk factors.

## Introduction

Colorectal cancer (CRC) has become the third most common cancer worldwide [1]. Stage II disease (T3N0M0 or T4N0M0) is diagnosed in approximately one-third of the patients with CRC [2] and the 5-year survival rate ranges from 44 to 93% [3]. Tumor local and distant recurrences are the main reasons for unfavorable outcomes for patients with CRC. Previous research showed that the distant recurrence rate was about 50% in patients undergoing oncologic resection for stage II and III CRC [4]. Although patients with stage II CRC are generally considered to have good prognoses after surgery, approximately 5–30% of these patients may develop local recurrence or metastasis after surgery [5, 6].

Adjuvant chemotherapy following surgery has been demonstrated to improve the overall survival (OS) and relapse free survival (RFS) in patients with stage III CRC. However, the role of adjuvant chemotherapy for stage II CRC remains controversial [7, 8]. At present, adjuvant chemotherapy is only recommended for stage II disease when high-risk factors are present. However, the definitions of high-risk factors vary among different guidelines [9–12]. Moreover, two recent randomized controlled trials in stage II CRC reported that there was no significant improvement in OS or RFS after adjuvant chemotherapy [13, 14]. All these evidences indicated that the present high-risk factors not always able to accurately predict the recurrence of stage II CRC patients [15].

Therefore, to obtain a clear understanding of the high-risk factors and identify the optimal selection of adjuvant chemotherapy, further investigation is warranted. The aim of the study was to investigate risk factors associated with postoperative recurrence in patients with stage II CRC undergoing curative surgery.

## Materials and methods

### Ethics statement

This study was approved by the Medical Ethics Committee of the Sixth Affiliated Hospital of Sun Yat-sen University, Guangzhou, China (no. 2021ZSLYEC-006).

### Inclusion and exclusion criteria

Patients who were diagnosed with pathologically confirmed stage II CRC according to the AJCC/UICC tumor-node-metastasis (TNM) system were identified from a prospectively maintained database at the Sixth Affiliated Hospital of Sun Yat-sen University between April 2008 to March 2019. Patients were excluded if they received preoperative radiotherapy or chemotherapy, and those who had more than one primary lesion, a pervious history of colorectal surgery, or insufficient follow-up information were also excluded.

### Demographic and clinical variables

Preoperative demographics and clinical records of all patients were reviewed to obtain information pertaining to age at surgery, sex, body mass index (BMI), family history, tumor diameter, pT-staging, lymphovascular invasion, perineural invasion, histological differentiation, tumor location, and postoperative chemotherapy. The morphological types of CRC specimens were evaluated by two experienced pathologists according to the Chinese Standard for Diagnosis and Treatment of Colorectal Cancer (2020). Any tumors whose main body protrudes into the intestinal lumen belong to Expansive type. Tumors that form deep ulcers or penetrate the muscle layer are classified as ulcerative type. The infiltrative type refers to the diffuse infiltration of the tumor into all layers of the intestinal wall, which thickens the local intestinal wall, but there is often no obvious ulcer or bulge on the surface. As one of the main clinical centers for CRC, the tumor marker levels (including carcinoembryonic antigen (CEA), carbohydrate antigen (CA) 199, CA125, and alpha fetoprotein (AFP)) were routinely tested within a month before the surgical intervention in our hospital. In additon, several studies have demostrated that these factors are closely related to the occurrence and development of gastrointestinal tumors [16–18]. So, these tumor marker levels were also collected in our study.

Postoperative follow-up was scheduled for surveillance every 3 months during the first year after the surgery, every 6 months during the next 2 years, and once a year thereafter. Recurrence was defined as the presence of radiologically confirmed or histologically proven tumor local or distant relapse in the follow-up period after surgical resection. OS was defined as the time from the first surgical resection to death from any cause.

### Standard and scheme of postoperative adjuvant chemotherapy

For patients with stage II colorectal cancer with traditional clinicopathological risk factors (pT4, lymphovascular invasion, high grade or poorly differentiated tumors, perforation or bowel obstruction, and < 12 lymph nodes examined), our center recommends postoperative neoadjuvant chemotherapy. Recommended chemotherapy regimens include: XELOX and mFOLFOX.

### Statistics analysis

The results of the descriptive data are presented as frequencies and percentages for categorical variables, or median and inter-quartile ranges for continuous variables. The two independent samples χ^2^ test or two-tailed Fisher’s exact test were used for categorical variables to compare the basic characteristics between patients with and without recurrence. The Kaplan–Meier method and log-rank test were performed to plot the survival curve and to compare the survival data. Univariate and multivariable Cox regression analyses were used to investigate the risk factors for recurrence. Differences were considered statistically significant when the *p* value was < 0.05. Variables with a *p* value of less than 0.05 in the univariate analysis were included in multivariable model. All analyses were performed using R Language for Statistical Computing (version 3.6.3).

## Results

### Patient characteristics

**Fig. 1:**
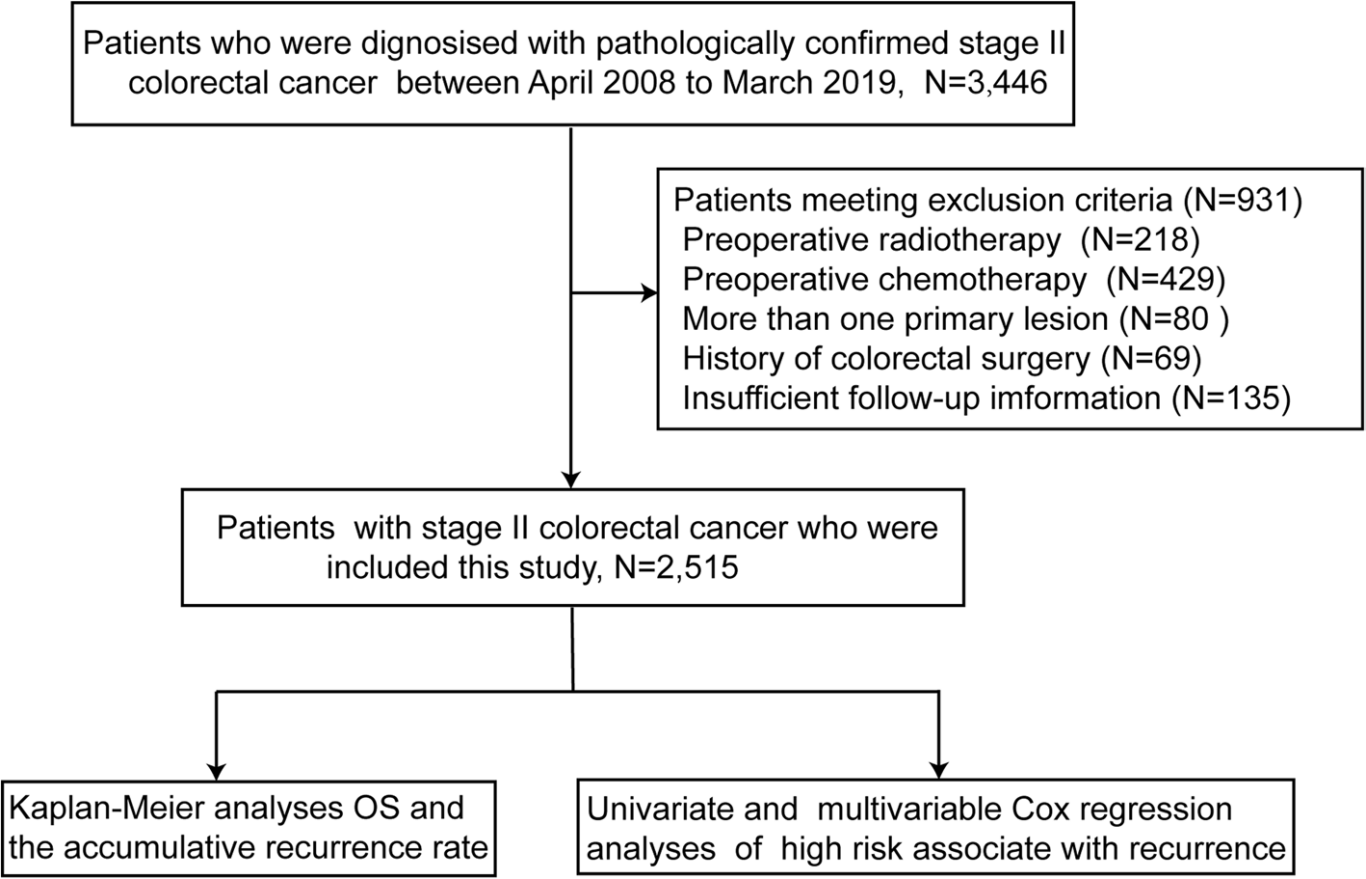
Flowchart of this study

As shown in Fig. 1, a total of 2515 patients who were diagnosed pathologically with stage II CRC were included in this study. Their clinicopathological characteristics are summarized in Table 1. The median age was 62 (52–70) years, and there were 1541 (61.3%) males. There were 579 (23.0%) patients with a BMI ≥ 25 kg/m^2^, and 74 (2.9%) patients had a family history of CRC. Tumor locations included right-sided colon cancer (*n* = 757, 30.1%), left-sided colon cancer (*n* = 885, 35.2%), and rectal cancer (*n* = 873, 34.7%).

**Table 1:** Clinical and pathological characteristics of stage II CRC patients

Characteristic	Overall (*n* = 2515)	Non-recurrenc (*n* = 2282)	recurrence (*n* = 233)	*P* Value
**Age**				0.142
< 60	1175 (46.7%)	1055 (46.2%)	120 (51.5%)	
≥ 60	1340 (53.3%)	1227 (53.8%)	113 (48.5%)	
**Sex**				0.418
male	1541 (61.3%)	1392 (61.0%)	149 (63.9%)	
female	974 (38.7%)	890 (39.0%)	84 (36.1%)	
**BMI**				0.608
< 25	1936 (77.0%)	1753 (76.8%)	183 (78.5%)	
≥ 25	579 (23.0%)	529 (23.2%)	50 (21.5%)	
**Tumor diameter**				** < 0.001**
< 5 cm	1743 (69.3%)	1617 (70.9%)	126 (54.1%)	
≥ 5 cm	772 (30.7%)	665 (29.1%)	107 (45.9%)	
**pT-staging**				**0.048**
T3	2295 (91.3%)	2091 (91.6%)	204 (87.6%)	
T4	220 (8.7%)	191 (8.4%)	29 (12.4%)	
**Lymphovascular invasion**				0.732
Negative	2390 (95.0%)	2167 (95.0%)	223 (95.7%)	
Positive	125 (5.0%)	115 (5.0%)	10 (4.3%)	
**Perineural invasion**				0.328
Negative	2294 (91.2%)	2086 (91.4%)	208 (89.3%)	
Positive	221 (8.8%)	196 (8.6%)	25 (10.7%)	
**CEA**				0.101
≤ 5 ng/ml	1620 (64.4%)	1458 (63.9%)	162 (69.5%)	
> 5 ng/ml	895 (35.6%)	824 (36.1%)	71 (30.5%)	
**CA199**				0.065
≤ 37 ng/ml	2215 (88.1%)	2019 (88.5%)	196 (84.1%)	
> 37 ng/ml	300 (11.9%)	263 (11.5%)	37 (15.9%)	
**CA125**				**0.025**
≤ 35 ng/ml	2340 (93.0%)	2132 (93.4%)	208 (89.3%)	
> 35 ng/ml	175 (7.0%)	150 (6.6%)	25 (10.7%)	
**AFP**				0.108
≤ 25 ng/ml	2476 (98.4%)	2250 (98.6%)	226 (97.0%)	
> 25 ng/ml	39 (1.6%)	32 (1.4%)	7 (3.0%)	
**Family History**				0.581
Negative	2441 (97.1%)	2213 (97.0%)	228 (97.9%)	
Positive	74 (2.9%)	69 (3.0%)	5 (2.1%)	
**Adjuvant chemotherapy**				**0.002**
Negative	1740 (69.2%)	1558 (68.3%)	182 (78.1%)	
Positive	775 (30.8%)	724 (31.7%)	51 (21.9%)	
**Differentiation**				0.115
High	586 (23.3%)	522 (22.9%)	64 (27.5%)	
Moderate	1712 (68.1%)	1568 (68.7%)	144 (61.8%)	
Poor	52 (2.1%)	44 (1.9%)	8 (3.4%)	
Undifferentital	16 (0.6%)	13 (0.6%)	3 (1.3%)	
Unknown	149 (5.9%)	135 (5.9%)	14 (6.0%)	
**Morphological type**				**0.015**
Expansive	827 (32.9%)	770 (33.7%)	57 (24.5%)	
Infiltrative	54 (2.1%)	49 (2.1%)	5 (2.1%)	
Ulcerative	1634 (65.0%)	1463 (64.1%)	171 (73.4%)	
**Location**				**0.025**
Right colon	757 (30.1%)	691 (30.3%)	66 (28.3%)	
Left colon	885 (35.2%)	817 (35.8%)	68 (29.2%)	
Rectum	873 (34.7%)	774 (33.9%)	99 (42.5%)	

Postoperative pathological examination showed that the proportions of pT3 and pT4 were 91.3% and 8.7%. There were 772 (30.7%) patients with a tumor diameter ≥ 5 cm, 125 (5.0%) patients with positive lymphovascular invasion, and 221 (8.8%) patients with positive perineural invasion.

### Characteristics of recurrence

A total of 233 (9.3%) patients developed postoperative tumor recurrence with 42 (1.7%) were local recurrence and 191 (7.6%) were distant metastasis. Sites of distant metastatic included the liver (*n* = 93, 3.7%), lung, bone, and brain metastases accounted for 2.5% (*n* = 63), 0.3% (*n* = 8) and 0.1% (*n* = 3) of all cases. Compared with the patients without recurrence, the patients with recurrence were significantly associated with increased tumor diameter (45.9% *vs*. 29.1%, *p* < 0.001), more pT4 tumors (12.4% *vs*. 8.4%, *p* = 0.048), and higher CA125 levels (10.7% *vs.* 6.6%, *p* = 0.025) (Table 1).

### Kaplan–Meier analyses of OS and the cumulative recurrence rate

**Fig. 2:**
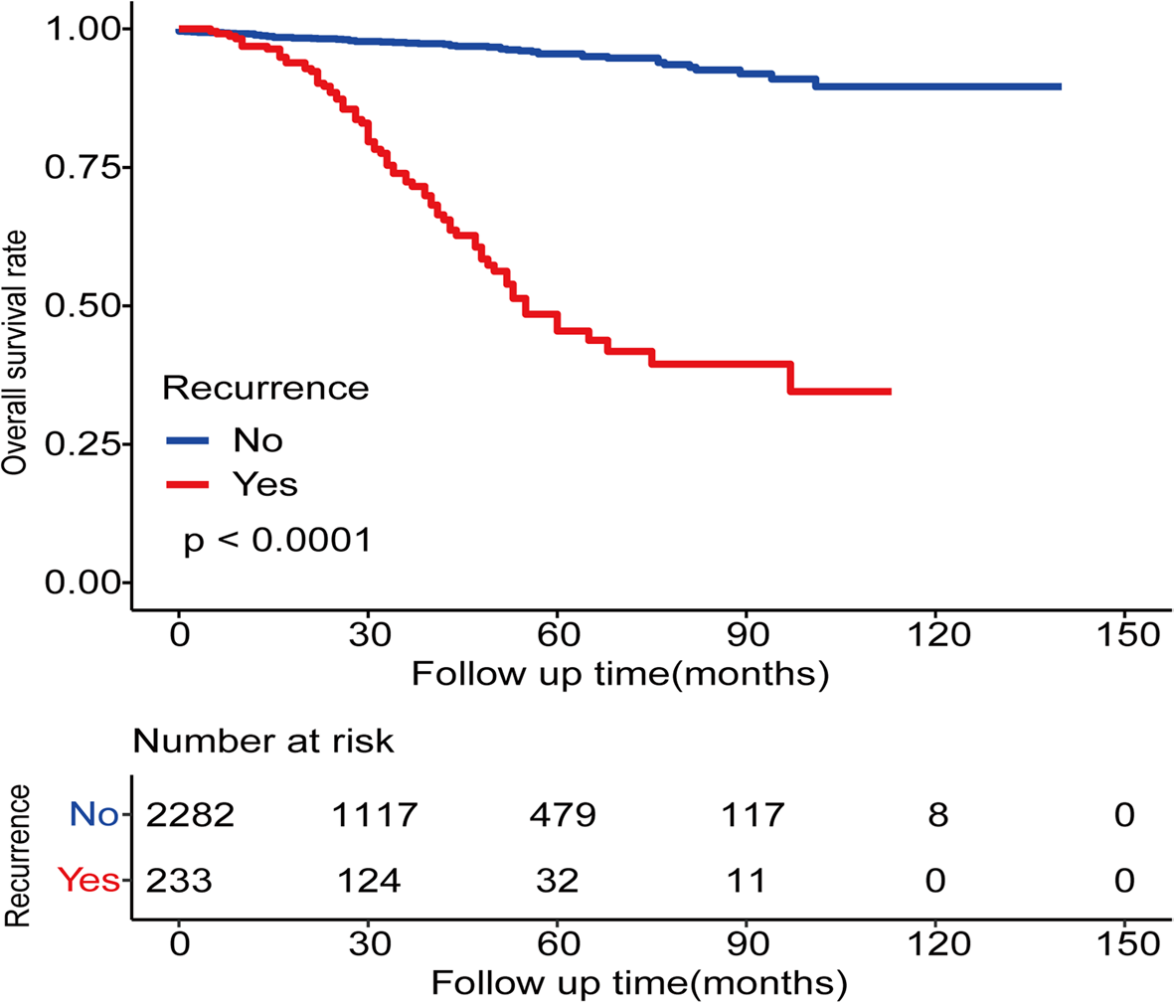
Kaplan–Meier analyses and risk table of OS. In stage II CRC patients, the OS was significant reduction in the recurrence patients compared with the non-recurrence patients (*p* < 0.0001)

**Fig. 3:**
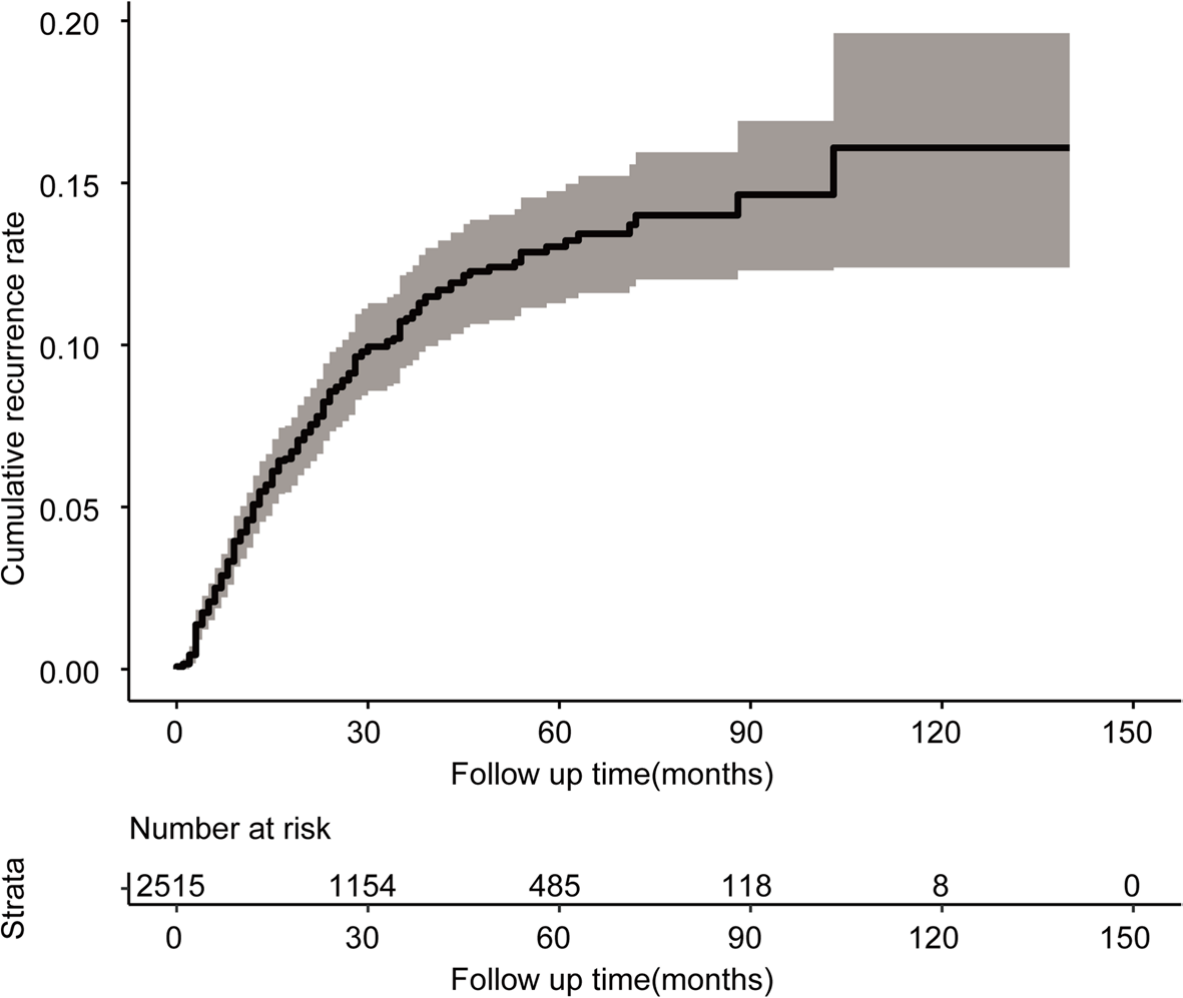
The cumulative recurrence rate of stage II CRC patients

Among all 2515 patients with stage II CRC, the Kaplan–Meier curves revealed significantly worse prognosis in patients with recurrence compared with those without (*p* < 0.0001) (Fig. 2). The 1-, 3-, and 5-year OS rate of the patients without recurrence were 98.9, 97.5, and 95.5%, and the 1-, 3-, and 5-year OS rate of patients with recurrence were 96.9, 72.4, and 45.4% (Table 2). As shown in Fig. 3, the 5-year cumulative recurrence rate for the patients with stage II CRC was 13%.

**Table 2:** The 1-, 3-, and 5-year OS of patients with stage II CRC

	**Non-recurrenc** **(*n* = 2282)**	**Recurrence** **(*n* = 233)**
1-year OS rate	98.9%	96.9%
3-year OS rate	97.5%	72.4%
5-year OS rate	95.5%	45.4%

### Risk factors for postoperative recurrence

After univariate Cox analyses, tumor diameter, pT-staging, preoperative CA199 level, preoperative CA125 level, postoperative chemotherapy and tumor morphological type were selected for multivariable Cox analysis (*p* < 0.05) (Table 3). Multivariable Cox analyses revealed that tumor diameter, preoperative CA125 level, preoperative CA199 level, and tumor morphological type were independent risk factors for postoperative recurrence in patients with stage II CRC (*p* < 0.05). As shown in Table 4, patients with a tumor diameter ≥ 5 cm, preoperative CA125 > 35 ng/ml, preoperative CA199 > 5 ng/ml, and ulcerating tumor had 1.79, 1.78, 1.56, and 1.61-fold increase in the risk of recurrence, respectively, compared with patients without recurrence.

**Table 3:** Univariate Cox regression analyses of factors associated with recurrence

Variable	HR (95% CI)	*P* Value
Age	0.83 (0.64–1.07)	0.150
Sex	0.88 (0.67–1.15)	0.333
BMI	0.90 (0.66–1.23)	0.495
Tumor diameter	1.86 (1.44–2.41)	** < 0.001**
pT-staging	1.50 (1.02–2.22)	**0.039**
Lymphovascular invasion	0.91 (0.48–1.71)	0.764
Perineural invasion	1.45 (0.96–2.20)	0.081
CEA	0.82 (0.62–1.09)	0.175
CA199	1.63 (1.15–2.32)	**0.006**
CA125	1.87 (1.2–2.8)	**0.003**
AFP	2.07 (0.98–4.39)	0.057
Family History	0.71 (0.29–1.73)	0.456
Adjuvant chemotherapy	0.72 (0.52–0.98)	**0.034**
Differentiation	1.02 (0.90–1.20)	0.540
Morphological type	1.2 (1.1–1.4)	**0.005**
Location	1.10 (0.90–1.21)	0.480

**Table 4:** Multivariable Cox regression analyses of factors influencing recurrence

Variable	HR (95% CI)	*P* Value
Tumor diameter (ref. = < 5 cm)	1.79 (1.38, 2.33)	** < 0.001**
CA125 (ref. = ≤ 35 ng/ml)	1.78 (1.17, 2.70)	**0.007**
CA199 (ref. = ≤ 5 ng/ml)	1.56 (1.09, 2.22)	**0.014**
pT-staging (ref. = T3)	1.25 (0.84, 1.86)	0.266
Morphological type (ref. = Expansive)		
Infiltrative	1.67 (0.66–4.21)	0.276
Ulcerative	1.61 (1.19–2.17)	**0.002**
Adjuvant chemotherapy (ref. = Negative)	0.71 (0.52, 0.97)	**0.029**

### Influence of adjuvant chemotherapy on recurrence

**Fig. 4:**
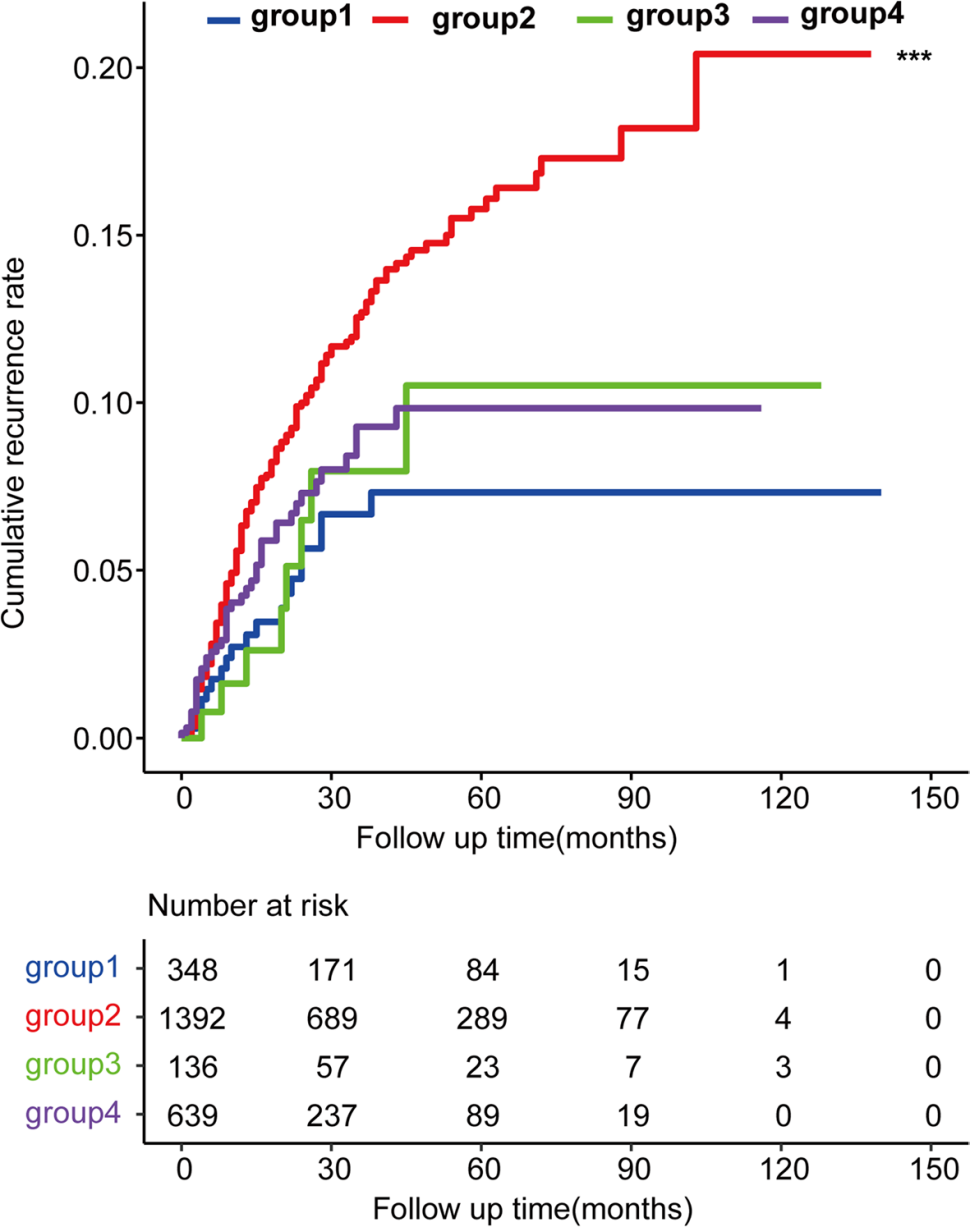
The cumulative recurrence rates of subgroups patients. Group 1: patients without risk factors and adjuvant chemotherapy; group 2: patients with one or more risk factors and without adjuvant chemotherapy; group 3: patients underwent adjuvant chemotherapy and without risk factors; group 4: patients with one or more risk factors and underwent adjuvant chemotherapy. ***: *p* = 0.00096 (group 2 vs. group 4)

As shown in the multivariable Cox regression analyses, patients receiving adjuvant chemotherapy were associated with a lower risk of recurrence (Hazard Ratio (HR): 0.71, 95% confidence interval (CI): 0.52–0.97). We performed a Kaplan–Meier stratified analysis of the cumulative recurrence rate with patients separated into four groups (group 1: Patients without risk factors and adjuvant chemotherapy; group 2: Patients with one or more risk factors and without adjuvant chemotherapy; group 3: Patients who received adjuvant chemotherapy but were without risk factors; group 4: Patients with one or more risk factors who underwent adjuvant chemotherapy). As shown in Fig. 4, adjuvant chemotherapy reduced the cumulative recurrence rate significantly in patients with risk factors of recurrence (group 4 *vs*. group 2, *p* = 0.00096). The 5-year cumulative recurrence rate was 9.8% in group 4, and 15.8% in group 2.

## Discussion

Recurrence, including local and distant recurrence, is the main reason for unfavorable outcomes in patients with stage II CRC. As reported by previous studies, the cumulative post-surgical recurrence rate was 5–30% in patients with stage II CRC [5, 6]. The current study analyzed 2515 patients with stage II CRC and reported that the 5-year cumulative recurrence rate for these patients was 13%. Patients with recurrence had a significantly worse 5-year OS compared with that in patients without recurrence (45.4% *vs.* 95.5%, *p* < 0.0001). These results revealed that there was a high rate of recurrence in patients with stage II CRC, despite the performance of radical surgery, and that recurrence was an important factor associated with poor prognosis. Therefore, subsequent treatment after surgery may be warranted in the patients with potential risk of recurrence.

To date, it has been difficult to identify exactly those patients at high-risk of recurrence. Thus, individual postoperative therapeutic strategies cannot be proposed exactly. High-risk factors in stage II CRC traditionally included pT4, lymphovascular invasion, high grade or poorly differentiated tumors, perforation or bowel obstruction, and < 12 lymph nodes examined [19, 20]. Although the ASCO, NCCN, and ESMO presented these high-risk factors for stage II CRC and suggested that clinicians consider adjuvant chemotherapy for patients with one or more of these high-risk factors [9, 10, 21], recent studies showed that survival outcome was influenced only by some of the known prognostic factors, and might be affected by other factors not suggested in the guidelines [20, 22–24]. Recently, Bockelman et al. [25] reported that emergency surgery and the MMR status were confirmed as new risk factors. With a focus on tumor recurrence, we used univariate and multivariable Cox regression analyses to investigate the risk factors of recurrence and identified three new high-risk factors of recurrence in patients with stage II CRC, including a tumor diameter ≥ 5 cm, preoperative CA125 > 35 ng/ml, preoperative CA199 > 5 ng/ml, and an ulcerative tumor. Several studies found tumor size was a strong independent risk prognosis factors for patients with CRC [26–28]. Compared with small tumors, tumors with large diameters have a relatively worse prognosis. CA125 plays a crucial role in tumor cell growth, advancing tumorigenesis and metastases [29]. Patients with high serum levels of CA125 are significantly with a poor prognosis in colorectal peritoneal carcinomatosis. And compared with CEA, CA125 is a better predictive marker for predicting peritoneal dissemination of CRC [18, 30]. In addition, some research also found CA125 concentration has an association with liver metastasis of CRC [31, 32]. CA199 is a well-known tumor marker that has been extensively studied in various types of cancer, including CRC. Several studies have suggested that elevated levels of CA199 are associated with poor prognosis in patients with CRC. Furthermore, CA199 has been shown to have potential predictive value for CRC recurrence and survival after surgery [32]. Thus these new factors might be beneficial to further individualize postoperative therapeutic trategies. Lymphovascular invasion, although associated with tumor recurrence or metastasis, is considered a prognostic factor for breast and prostate cancer [33, 34]. However, the effect of lymphovascular infiltration on the recurrence or prognosis of CRC remains controversial. Masahiro Kataoka et al. demostrated that the value of lymphovascular invasion as a prognostic factor for stage II CRC [35]. While other investigators have not been able to determine any effect of vascular invasion on prognosis [36, 37]. In this study, we also found that lymphovascular invasion was not an independent prognostic factor for stage II colorectal cancer. Such difference may be due to different population or analysis methods.

Adjuvant chemotherapy is recommended in patients with stage III CRC because it can improve OS and RFS [38]. However, the role of adjuvant chemotherapy for patients with stage II CRC has been controversial [8, 39]. In patients with stage II CRC, there was a wide range of postoperative 5-year OS because heterogeneity existed among the patients with stage II CRC in terms of recurrence. Therefore, it is necessary to stratify at-risk patients according to possible risk factors to determine whether they might benefit from adjuvant chemotherapy [40]. In the present study, we separated patients into four groups based on above mentioned three high-risk factors and adjuvant chemotherapy. We found that adjuvant chemotherapy significantly reduced the cumulative recurrence rate in patients with these high-risk factors (*p* = 0.00096). In contrast, adjuvant chemotherapy showed no benefit for patients without these three risk factors. This indicated that these three new risk factors could be used effectively to identify patients that might benefit from adjuvant chemotherapy in stage II CRC.

There were several limitations to the present study. First, the study was a single-center retrospective study and selection bias is inevitable. Second, some patients did not have enough follow-up time, which might affect the accuracy of the results. Third, because of insufficient information, there remain some clinicopathological characteristics and genomic-based prognostic factors that were not included in this study. Therefore, longer follow-up and larger multi-center prospective studies are awaited to integrate more factors in a search for a more tailored prognostication of stage II CRC.

## Conclusion

In conclusion, a tumor diameter ≥ 5 cm, preoperative CA125 > 35 ng/ml, preoperative CA199 > 5 ng/ml, and an ulcerative tumor were high-risk factors for postoperative recurrence of stage II CRC. As a favorable prognostic factor of recurrence, adjuvant chemotherapy should be considered in patients with stage II CRC with these high-risk factors.

## Data Availability

The data sets generated and/or analyzed during the current study are available from the corresponding author upon reasonable request.

## Abbreviations

CRC: Colorectal cancer
OS: Overall survival
HR: Hazard Ratio
CA: Carbohydrate antigen
RFS: Relapse free survival
BMI: Body mass index
CEA: Carcinoembryonic antigen
AFP: Alpha fetoprotein
CI: Confidence interval

## References

[1] Bray F, Ferlay J, Soerjomataram I, Siegel RL, Torre LA, Jemal A (2018). Global cancer statistics 2018: GLOBOCAN estimates of incidence and mortality worldwide for 36 cancers in 185 countries. CA Cancer J Clin.

[2] Figueredo A, Charette ML, Maroun J, Brouwers MC, Zuraw L (2004). Adjuvant therapy for stage II colon cancer: a systematic review from the Cancer Care Ontario Program in evidence-based care’s gastrointestinal cancer disease site group. J Clin Oncol.

[3] O’connell JB, Maggard MA, Ko CY (2004). Colon cancer survival rates with the new American Joint Committee on Cancer sixth edition staging. J Natl Cancer Inst.

[4] Van Cutsem E, Oliveira J (2009). Advanced colorectal cancer: ESMO clinical recommendations for diagnosis, treatment and follow-up. Ann Oncol.

[5] Park JS, Chon HJ, Jeung H-C, Shin SJ, Rha SY, Ahn JB, Lee KY, Kim NK, Chung HC (2016). High-risk clinicopathological features and their predictive significance in Korean patients with stage II colon cancer. J Cancer Res Clin Oncol.

[6] Law WL, Ho JWC, Chan R, Au G, Chu KW (2005). Outcome of anterior resection for stage II rectal cancer without radiation: the role of adjuvant chemotherapy. Dis Colon Rectum.

[7] Okuda Y, Shimura T, Yamada T, Hirata Y, Yamaguchi R, Sakamoto E, Kataoka H (2018). Colorectal obstruction is a potential prognostic factor for stage II colorectal cancer. Int J Clin Oncol.

[8] Gray R, Barnwell J, McConkey C, Hills RK, Williams NS, Kerr DJ (2007). Adjuvant chemotherapy versus observation in patients with colorectal cancer: a randomised study. Lancet.

[9] Benson AB, Schrag D, Somerfield MR, Cohen AM, Figueredo AT, Flynn PJ, Krzyzanowska MK, Maroun J, McAllister P, Van Cutsem E (2004). American society of clinical oncology recommendations on adjuvant chemotherapy for stage II colon cancer. J Clin Oncol.

[10] Labianca R, Nordlinger B, Beretta GD, Mosconi S, Mandalà M, Cervantes A, Arnold D (2013). Early colon cancer: ESMO Clinical Practice Guidelines for diagnosis, treatment and follow-up. Ann Oncol.

[11] Benson AB, Venook AP, Al-Hawary MM, Cederquist L, Chen Y-J, Ciombor KK, Cohen S, Cooper HS, Deming D, Engstrom PF (2018). NCCN guidelines insights: colon cancer, version 2.2018. J Natl Compr Canc Netw.

[12] Benson AB, Venook AP, Al-Hawary MM, Arain MA, Chen Y-J, Ciombor KK, Cohen S, Cooper HS, Deming D, Garrido-Laguna I (2020). NCCN guidelines insights: rectal cancer, version 6.2020. J Natl Compr Canc Netw.

[13] Glimelius B, Dahl O, Cedermark B, Jakobsen A, Bentzen SM, Starkhammar H, Grönberg H, Hultborn R, Albertsson M, Påhlman L (2005). Adjuvant chemotherapy in colorectal cancer: a joint analysis of randomised trials by the Nordic Gastrointestinal Tumour Adjuvant Therapy Group. Acta Oncol.

[14] Schippinger W, Samonigg H, Schaberl-Moser R, Greil R, Thödtmann R, Tschmelitsch J, Jagoditsch M, Steger GG, Jakesz R, Herbst F (2007). A prospective randomised phase III trial of adjuvant chemotherapy with 5-fluorouracil and leucovorin in patients with stage II colon cancer. Br J Cancer.

[15] Dotan E, Cohen SJ (2011). Challenges in the management of stage II colon cancer. Semin Oncol.

[16] Ren J, Xu L, Zhou S, Ouyang J, You W, Sheng N, Yan L, Peng D, Xie L, Wang Z (2021). Clinicopathological features combined with immune infiltration could well distinguish outcomes in stage ii and stage III colorectal cancer: a retrospective study. Front Oncol.

[17] Luo H, Shen K, Li B, Li R, Wang Z, Xie Z (2020). Clinical significance and diagnostic value of serum NSE, CEA, CA19-9, CA125 and CA242 levels in colorectal cancer. Oncol Lett.

[18] Huang C-J, Jiang J-K, Chang S-C, Lin J-K, Yang S-H (2016). Serum CA125 concentration as a predictor of peritoneal dissemination of colorectal cancer in men and women. Medicine (Baltimore).

[19] Ishiguro M, Ueno H, Kanemitsu Y, Hamaguchi T, Shida D, Shimada Y (2018). Current clinical practice of adjuvant chemotherapy for patients with ‘high-risk’ Stage II colorectal cancer in Japan: a questionnaire survey in the JCOG Study Group. Jpn J Clin Oncol.

[20] Verhoeff SR, van Erning FN, Lemmens VEPP, de Wilt JHW, Pruijt JFM (2016). Adjuvant chemotherapy is not associated with improved survival for all high-risk factors in stage II colon cancer. Int J Cancer.

[21] André T, Boni C, Mounedji-Boudiaf L, Navarro M, Tabernero J, Hickish T, Topham C, Zaninelli M, Clingan P, Bridgewater J (2004). Oxaliplatin, fluorouracil, and leucovorin as adjuvant treatment for colon cancer. N Engl J Med.

[22] Kumar A, Kennecke HF, Renouf DJ, Lim HJ, Gill S, Woods R, Speers C, Cheung WY (2015). Adjuvant chemotherapy use and outcomes of patients with high-risk versus low-risk stage II colon cancer. Cancer.

[23] Lin H-H, Chang Y-Y, Lin J-K, Jiang J-K, Lin C-C, Lan Y-T, Yang S-H, Wang H-S, Chen W-S, Lin T-C (2014). The role of adjuvant chemotherapy in stage II colorectal cancer patients. Int J Colorectal Dis.

[24] Quah H-M, Chou JF, Gonen M, Shia J, Schrag D, Landmann RG, Guillem JG, Paty PB, Temple LK, Wong WD (2008). Identification of patients with high-risk stage II colon cancer for adjuvant therapy. Dis Colon Rectum.

[25] Böckelman C, Engelmann BE, Kaprio T, Hansen TF, Glimelius B (2015). Risk of recurrence in patients with colon cancer stage II and III: a systematic review and meta-analysis of recent literature. Acta Oncol.

[26] Maniwa T, Mori K, Ohde Y, Okumura T, Boku N, Hishida T, Sakao Y, Yoshiya K, Hyodo I, Kondo H (2017). Heterogeneity of tumor sizes in multiple pulmonary metastases of colorectal cancer as a prognostic factor. Ann Thorac Surg.

[27] Kornprat P, Pollheimer MJ, Lindtner RA, Schlemmer A, Rehak P, Langner C (2011). Value of tumor size as a prognostic variable in colorectal cancer: a critical reappraisal. Am J Clin Oncol.

[28] Kato T, Alonso S, Muto Y, Perucho M, Rikiyama T (2016). Tumor size is an independent risk predictor for metachronous colorectal cancer. Oncotarget.

[29] Thériault C, Pinard M, Comamala M, Migneault M, Beaudin J, Matte I, Boivin M, Piché A, Rancourt C (2011). MUC16 (CA125) regulates epithelial ovarian cancer cell growth, tumorigenesis and metastasis. Gynecol Oncol.

[30] Huo YR, Huang Y, Liauw W, Zhao J, Morris DL (2016). Prognostic Value of Carcinoembryonic Antigen (CEA), AFP, CA19-9 and CA125 for Patients with Colorectal Cancer with Peritoneal Carcinomatosis Treated by Cytoreductive Surgery and Intraperitoneal Chemotherapy. Anticancer Res.

[31] Mavligit GM, Estrov Z (2000). CA 125: a clinically useful tumor marker in the management of colorectal carcinoma metastatic to the liver in patients with normal carcinoembryonic antigen. Am J Clin Oncol.

[32] Zhang D, Yu M, Xu T, Xiong B (2013). Predictive value of serum CEA, CA19-9 and CA125 in diagnosis of colorectal liver metastasis in Chinese population. Hepatogastroenterology.

[33] Aleskandarany MA, Sonbul SN, Mukherjee A, Rakha EA (2015). Molecular mechanisms underlying lymphovascular invasion in invasive breast cancer. Pathobiology.

[34] Jeong J-U, Nam T-K, Song J-Y, Yoon MS, Ahn S-J, Chung W-K, Cho IJ, Kim Y-H, Cho SH, Jung SI (2019). Prognostic significance of lymphovascular invasion in patients with prostate cancer treated with postoperative radiotherapy. Radiat Oncol J.

[35] Kataoka M, Hirano Y, Ishii T, Kondo H, Asari M, Ishikawa S, Kataoka A, Fujii T, Shimamura S, Yamaguchi S (2021). Impact of lymphovascular invasion in patients with stage II colorectal cancer: a propensity score-matched study. In Vivo.

[36] Minsky BD, Mies C, Recht A, Rich TA, Chaffey JT (1988). Resectable adenocarcinoma of the rectosigmoid and rectum. II. The influence of blood vessel invasion. Cancer.

[37] Artac M, Turhal NS, Kocer M, Karabulut B, Bozcuk H, Yalcin S, Karaagac M, Gündüz S, Isik N, Uygun K (2014). Do high-risk features support the use of adjuvant chemotherapy in stage II colon cancer?. A Turkish Oncology Group study Tumori.

[38] Sobrero AF, Puccini A, Shi Q, Grothey A, Andrè T, Shields AF, Souglakos I, Yoshino T, Iveson T, Ceppi M (2020). A new prognostic and predictive tool for shared decision making in stage III colon cancer. Eur J Cancer.

[39] Tournigand C, André T, Bonnetain F, Chibaudel B, Lledo G, Hickish T, Tabernero J, Boni C, Bachet J-B, Teixeira L (2012). Adjuvant therapy with fluorouracil and oxaliplatin in stage II and elderly patients (between ages 70 and 75 years) with colon cancer: subgroup analyses of the Multicenter International Study of Oxaliplatin, Fluorouracil, and Leucovorin in the Adjuvant Treatment of Colon Cancer trial. J Clin Oncol.

[40] Rebuzzi SE, Pesola G, Martelli V, Sobrero AF (2020). Adjuvant chemotherapy for stage II colon cancer. Cancers (Basel).

